# ‘You just eyeball it’: Parent and nursery staff perceptions and influences on child portion size: A reflexive thematic analysis

**DOI:** 10.1177/02601060241245255

**Published:** 2024-04-16

**Authors:** Sophia Quirke-McFarlane, Sharon A Carstairs, Joanne E Cecil

**Affiliations:** 1 Population and Behavioural Sciences, School of Medicine, 7486 University of St Andrews, St Andrews, UK

**Keywords:** Portion size, preschool children, caregivers, qualitative, thematic analysis

## Abstract

**Background:** Childhood obesity is one of the most serious public health epidemics of the 21st century. Observational studies report that increases in portion size (PS) have occurred in parallel with levels of obesity. Increased PSs of high-energy-dense foods can promote overeating, and without compensatory behaviours, can contribute to childhood obesity. Caregivers make decisions about PSs for children in the home and nursery environment, thus are gatekeepers to child food intake. Understanding caregiver PS decisions can aid in the best practice of PS provision to young children. The aim of this study was to explore parent and nursery staff influences on child PS selection and their suggestions for useful tools/strategies in PS decisions. **Methods:** A qualitative design was employed using focus group discussions (FGDs) with parents and nursery staff of children aged 3–5 years. FGDs were employed given their ability to generate rich data, as well as permit the exploration of collective perceptions, attitudes, behaviours and experiences. Data were analysed using an inductive, semantic approach to reflexive thematic analysis. **Results:** Four FGDs were conducted: two with parents (*n *= 13), two with nursery staff (*n *= 17). Four overarching themes were derived: (i) awareness of PS guidelines; (ii) control over PS; (iii) social influences on children's eating behaviours; (iv) child-specific, social and external factors influencing parent and nursery staff PS decisions. Additionally, participants discussed tools/strategies they believe would be useful in PS decisions. **Conclusion:** Data from the themes suggest that caregiver control, social, child-specific and external factors are more influential than PS guidelines in both parent and nursery staff PS decisions for young children aged 3–5 years. These findings can inform future childhood obesity prevention initiatives focussed on improving parent and nursery staff provision/use of age-appropriate PSs.

## Introduction

Childhood obesity is one of the greatest public health epidemics of the 21st century (World Health Organization, 2021). Global prevalence for children up to 5 years presenting with overweight and obesity was 38.2 million in 2019 (World Health Organization, 2021). In Scotland, 18% of children aged 2–15 years were at risk of obesity in 2021 (The Scottish Government, 2022). Obesity has been associated with comorbidities including cardiovascular disease and diabetes and has been identified as a major risk factor for mortality and morbidity (Hruby et al., 2016). Research suggests that childhood obesity is highly predictive of obesity in adulthood (Geserick et al., 2018; Simmonds et al., 2016), thus early intervention is critical in obesity prevention and early childhood provides a key period for the promotion of healthy dietary habits that continue into later life stages (Schwartz et al., 2011).

Increased portion sizes (PSs) of high-energy-dense (HED) foods are a significant factor contributing to overconsumption (Ello-Martin et al., 2005; Flieh et al., 2021; Rolls et al., 2007). Since the 1970s, sizes of HED food portions, serving utensils and tableware have increased considerably (Piernas and Popkin, 2011). Observational studies show that increased PSs of HED foods have occurred in parallel with levels of obesity (Hales et al., 2018; Livingstone and Pourshahidi, 2014; Piernas and Popkin, 2011). Although a causal relationship cannot be demonstrated, findings suggest that increased PSs promote overconsumption (Ello-Martin et al., 2005; Rolls et al., 2007) and, without later compensation, can modify perceptions of PS norms (Herman and Polivy, 2005) and contribute to obesity (Hales et al., 2018; Hetherington et al., 2018; Piernas and Popkin, 2011; Rolls, 2003; Smethers et al., 2019). Experimental studies conducted in children support this association, demonstrating a greater volume of food is consumed when larger portions are presented compared with smaller portions (Ello-Martin et al., 2005; Fisher et al., 2003; Fisher and Kral, 2008; Rolls et al., 2000; Carstairs et al., 2018), a phenomenon coined as the ‘portion size effect’. In their recent review, Zuraikat et al. (2019) identified environmental factors, food-related factors and consumer characteristics as potential moderators of the PSE, highlighting the complex interplay between moderators underlying this.

In the context of child food providers, research has traditionally focused on parental feeding/portioning practices (Birch, 2006; Birch et al., 2003; Rolls et al., 2000; Sherry et al., 2004). However, as more children attend full-time non-parental childcare (Care Inspectorate, 2022), due to factors such as the increase in mothers joining the workforce (Office for National Statistics, 2022) and Governmental financial support (GOV.UK, 2023), more children are consuming a large proportion of their daily meals in these environments (Gubbels et al., 2010; Larson et al., 2011). Therefore, the feeding practices implemented by nursery staff have become a focus of interest (Benjamin Neelon et al., 2012; Dev et al., 2016; Larson et al., 2011; Love et al., 2020; Ramsay et al., 2010; Wallace and Mills, 2019). Nursery staff may provide different insights and perspectives on child feeding practices and PS provision than parents due to the out of home environment that the childcare setting provides, and possibly due to institutional training and guidelines they might be required to undertake in their role. Research on pre-school childcare staff (Dev et al., 2016; Ramsay et al., 2010) found that some staff members use controlling feeding practices (e.g. pressure to eat) to encourage children to eat, verbally cueing children to eat in response to external cues (e.g. food) rather than internal cues (e.g. satiety). More recently, Wallace and Mills (2019) found that 20% of family day-care educators lacked confidence in estimating the recommended serving of food for children. Together, these data highlight that parents and nursery staff are important targets for the provision of age-appropriate PSs and encouraging healthy PS norms in children.

Research on parental PS practices suggests parents give young children greater PSs than physiologically required (Infant & Toddler Forum, 2014; Johnson et al., 2014; Nicklas et al., 2013; Reale et al., 2019b), for reasons such as fear of disordered growth and development (Shloim et al., 2015), desire not to waste food (Momin et al., 2014), limited/confused knowledge surrounding age-appropriate portions (Croker et al., 2009; Curtis et al., 2017; Douglas et al., 2014; McCrickerd and Forde, 2016; Reale et al., 2019a; Tang et al., 2020), as well as parents’ own hunger levels (Reale et al., 2019b). Qualitative studies on PS suggest some parents do not follow PS guidelines but use other means to determine the PS they serve children, such as children's appetitive characteristics (Carnell et al., 2011; Croker et al., 2009; Eck et al., 2018; Jacquier et al., 2016; Johnson et al., 2015; Reale et al., 2019a; Sherry et al., 2004), child-sized tableware (Curtis et al., 2017; Eck et al., 2018; Jacquier et al., 2016; Reale et al., 2019a; Tang et al., 2020), food type (Blake et al., 2015; Carnell et al., 2011; Croker et al., 2009; Eck et al., 2018; Fisher et al., 2015; Jacquier et al., 2016; Petrunoff et al., 2014), as well as caregivers own intuition (Blake et al., 2015; Sherry et al., 2004). However, most of the qualitative evidence-base has focused on parental caregiver decisions regarding child PSs and has not considered the views of other important caregiver groups, such as nursery staff. Understanding both parent and nursery staff portioning practices is warranted to aid in the best practice of caregiver portion provision to young children.

Using a qualitative approach, this study aimed to explore parent and nursery staff perceptions and influences on their portion selection and provision for the young children in their care, and to explore any tools and strategies they believe could be helpful in PS decisions.

## Materials and methods

### Research design

A qualitative design using focus group discussions (FGDs) was employed to explore participants’ perceptions and influences on child PS. FGDs are effective in generating detailed qualitative data, and permit the exploration of collective perceptions, attitudes, behaviours and experiences (Kitzinger, 1995). Compared with other qualitative data collection methods, FGDs can illuminate agreement and inconsistencies within and between groups (Goodman and Evans, 2015), making them appropriate for this study. The FGDs were designed to range between 30 and 45 minutes and included the use of a discussion topic guide (Supplemental Table 1). Key topics included influences on the determination of child PS, awareness and perceptions of PS guidelines, as well as prompts about tools/strategies to assist in the determination of child PS. All FGDs were audio-recorded. Parent FGDs were conducted in private meeting rooms on a university campus; nursery staff FGDs were conducted within local nursery settings (N.E. Scotland) after working hours.

### Participants and recruitment

Eligible participants were parents of children aged 3–5 years and nursery staff from local nurseries with children aged 3–5 years in their care. Parents and nursery staff were recruited via advertisements, university newsletters and social media. Participants received a £10 voucher recompense. Study procedures were reviewed and approved by the university's teaching and research ethics committee (MD12354).

### Data analysis

Audio-recorded FGDs were transcribed verbatim by a transcription service (Way with Words). One reviewer (SQM) examined the transcripts for accuracy by comparing the audio-recordings against the transcripts and removing all identifying information.

Given the exploratory nature of this study, the FGDs were analysed using an inductive, semantic approach to reflexive thematic analysis (TA) (Braun and Clarke, 2006, 2019). Reflexive TA involves identifying and analysing themes (patterns of meaning) within the raw data (Braun and Clarke, 2006, 2019). An inductive approach was employed to allow the identification of themes to occur from the data. This approach is appropriate due to its flexible nature and ability to provide a rich and detailed account of the data (Braun and Clarke, 2006, 2019). Braun and Clarke's (2006) six-step reflexive TA framework was followed. The reflexive TA coding was conducted manually and led by one researcher (SQM), following the guidelines of Clarke and Braun (2013). Two researchers (SC, JC) reviewed and approved the identified codes, subthemes and themes through independent review of the transcripts and ongoing discussion. Data collection continued until data saturation was reached (i.e. the point at which no new themes were identified).

## Results

Four FGDs were conducted, two with parents (FGD 2 *n *= 7; FGD 4 *n *= 6) and two with nursery staff (FGD 1 *n *= 12; FGD 3 *n *= 5). Data were collected between July 2018 and July 2019. The parent and nursery staff sample included 13 (10 females, 3 males) and 17 (16 females, 1 male) participants, respectively. Four overarching themes were derived to capture parental and nursery staff perceptions/influences about PS selection and provision: (i) awareness of PS guidelines; (ii) control over PS; (iii) social influences on child's eating behaviour; and (iv) child-specific, social and external factors influencing parent and nursery staff PS decisions (Supplemental Figure 1). Several parents and nursery staff provided suggestions for tools/strategies they believe would be useful in PS decisions.

### Factors influencing parent and nursery staff Ps selection and provision

#### Theme 1: Awareness of PS guidelines

##### Aware of PS guidelines

Some parents were aware of the existence of published PS guidelines for children, with one parent believing it would be difficult to be unaware of such guidelines due to their widespread media coverage (Table 1; Quotation number I). Discussions revolved around visual representations of portions or weighing of food items. There was agreement amongst some parents regarding the usefulness of visual PS guidelines (Table 1; QII). Although some parents were aware of PS guidelines (Table 1; QIII), they admitted disregarding them as weighing food was deemed too effortful (Table 1; QIV).

**Table 1.** Supporting quotations: theme ‘Awareness of portion size guidelines’.

Subtheme	Quotation no.	Supporting quotation
*Aware of portion size guidelines*	I	Yes, I mean it's [child portion size guidelines] in your face all the time whenever you put the television on or, you know, you go to the supermarket. It's in your face all the time. […] It's there, you know, every time you blink […] I think subconsciously, you do take that on board. [Parent 5, F, FGD 2]
	II	And then when they [children] went into having powdered milk and stuff, it was, like, this is how much they should have, and there's clear guidelines and there are some very nice guidelines to show the size of a baby's stomach and just what that equates to. So you know when you’re thinking about food portion sizes, that you’ve kind of got that to think about. [Parent 5, F, FGD 2] I found that [newborn stomach size] visual very good, as well. Especially after weaning and that started, I found that visual really helpful. [Parent 2, F, FGD 2]
	III	I still have it [Healthy Start guidelines booklet] at home. I mean, I don't necessarily follow it, but I’m aware that it exists. [Parent 3, F, FGD 4]
	IV	It's [weighing] too much hassle. [Parent 2, F, FGD 4] There's no way. That's why you use the fist thing, because it's a very quick […] You know, you just eyeball it. [Parent 6, M, FGD 4]
*Unaware of portion size guidelines*	I	I’m not aware of any documentation that states this is how much a two-year-old should be getting, or… You know, I’ve never seen anything yet. This is, you know, you should have a third of your plate vegetables and stuff like that, but nothing that guidelines how much they’d actually be having. [Nursery staff 7, F, FGD 1]
	II	We just judge it [portion sizes] ourselves. We probably should have a wheel of what sugar they’re meant to have and what sugar is in each thing. So I guess there are things like that that the government recommend. [Nursery staff 4, F, FGD 3]
	III	We probably should [have guidance on portion size selection]. Because everybody's perception of what portion a size should have is probably different really. [Nursery staff 4, F, FGD 3]
	IV	It would be good to know for the guidelines of maybe what is a good standard. [Nursery staff 1, F, FGD 3]
	V	I think it's [portion size] such a hot topic nowadays isn’t it? And we have got the children at such a young age, it's their best start in life so we need to be clear on what we can do to contribute to that and I think any information we’ve got would be valuable. [Nursery staff 2, M, FGD 3]

##### Unaware of PS guidelines

Compared with parents, nursery staff were less aware of any published child PS guidelines (Table 1; QI, QII). Some nursery staff expressed positive attitudes towards the provision of further PS information, stating additional information would be beneficial given their role (Table 1; QIII-QV).

#### Theme 2: Control over PS

##### Restriction

Parents and nursery staff exerted considerable control over the quantity and type of food children consume, with some believing children need a cue to stop eating (Table 2; QI-QIII). Parents’ and nursery staff's perceived healthfulness of food determined the extent to which they exert control; where foods deemed ‘healthy’ are restricted less compared with foods deemed ‘unhealthy’ (Table 2; QIV, QV). To ensure equity across the children in their care, some nursery staff are strict with the provision of second helpings (Table 2; QVI).

**Table 2.** Supporting quotations: theme ‘Control over portion size’.

Subtheme	Quotation no.	Supporting quotation
*Restriction*	I	[…] we say, “have you had enough, or would you like something else?” […] they [children] stop and say, well, maybe I’ve had enough. Whereas if you put everything on their plate one by one, they will just eat it because it's there. [Nursery staff 9, F, FGD 1]
** **	II	If we have a child that comes back to the table, like, three times, we say oh it has to go around to all your friends or aren’t you feeling full? Trying to make them actually think about it, rather than just eating and eating and eating. [Nursery staff 5, F, FGD 1]
** **	III	And you also start getting a sense of their addictions as well. […] You know, if you give her anything sweet, she’ll ask for more, and she’ll just keep asking for more and she won’t stop. She doesn’t have a stop signal for sugar, and that worries me. [Parent 6, M, FGD 4]
** **	IV	I guess if I deem it to be a healthy snack, then they [children] can have this many oatcakes if they want. But if I deem it to be an unhealthy snack, then I would cap it. [Parent 6, M, FGD 4]
** **	V	It depends on what kind of snacks it was […] If it was chocolate cake and they [children] wanted to keep eating it, I would stop that. […] But, if it was crackers and cheese I would maybe be a bit more free with that. And fruit as well. [Nursery staff 5, F, FGD 3]
** **	VI	We might say, well we will see if anyone else wants more and then, if there is any left, you [children] can maybe get a third bowl of fruit or something like that. But if everyone is having seconds it's not fair for someone to miss out. [Nursery staff 1, F, FGD 3]
*No restriction*	I	Depending on the meal, I would plate up, like, the main part of the meal, like the chicken or whatever, and then we have a bowl of vegetables on the table, and they’d [the children] help themselves. And the general rule is you have to try a little bit of everything, but you can help yourself. [Parent 7, F, FGD 2]
** **	II	[…] when I get home with the kids. If [name of child] cooks with me, he’ll eat all of it. […] Because he's done it. But then if I cook the same thing and put it on a plate: oh no, I don’t want that. [Parent 2, F, FGD 2]
** **	III	[…] it's [portion size selection] much more independent. We focus more on independence. So encourage them [children] to cut their own food, pick what they want, come back and forth […] When before, we would just pick up the snack, put it on the table. [Nursery staff 4, F, FGD 1]
** **	IV	But just say it's spaghetti bolognaise and pasta, so they [children] would portion it themselves. We’ve been doing that for quite a while now, haven’t we? [Nursery staff 4, F, FGD 3]
** **	V	Some children like to have that control at snack time, independence. They go and help themselves to whatever they’re having. And they eat what they put on their plate, and they’ve chosen it. The majority of the time, they’re quite happy to sit and eat what they’ve picked. But if you put that on a plate for them and set that in front of them, like they don’t want that, because they’ve not been in control of it. [Nursery staff 1, F, FGD 1]
*Different portioning practices*	I	The less on the plate, I find, like, when I put less on it, he’ll [son] eat it. A big [plate] of it, he won’t eat it. Even if he likes it. [Nursery staff 4, F, FGD 1]
	II	To make everyone [the children in their care] feel equal, we would put, […] the same on everyone's plate. Again like less is more. So there wouldn’t be much on everyone's plates and they can choose if they would like more. [Nursery staff 1, F, FGD 3]
	III	So […] what we’re trying to do is for them [children] to take a little bit, not to waste, and they can always take more if they’re still hungry […] And also because they’re super-picky eaters, this way they feel like they have more control over what they’re eating, so it helps them challenge themselves. [Parent 4, F, FGD 4]
	IV	Sometimes [name of child] does want seconds, but quite often, he’ll just eat what's on his plate. So I’ve quite often put quite a big portion, because I know he might lose interest, and then I’d always rather he, like, left a bit than maybe got hungry a bit later and wanted a bit of toast before bed. So yes, so sometimes I am mindful that I’ve kind of got one shot to have him eat his dinner. [Parent 2, F, FGD 2]

##### No restriction

Most parents and nursery staff granted children autonomy over their PSs and food choices, with some allowing children to self-serve and be actively involved with cooking/food selection (Table 2; QI, QII). Some nursery staff acknowledged a recent shift in the childcare sector towards granting children greater autonomy over their PS and food choice (Table 2; QIII, QIV). Variation in how children respond to having control over their PS and food choice was noted by nursery staff (Table 2; QV).

##### Different portioning strategies

A common strategy for controlling PS identified by several parents and nursery staff was to provide children with smaller PSs, and, if still hungry, allow them to have a second helping (Table 2; QI-QIII). Providing a child with a large PS was a strategy used by parents as they are aware they have one opportunity to encourage their child to eat dinner (Table 2; QIV).

#### Theme 3: Social influences on children's eating behaviours

##### Peer influence

Some parents and nursery staff agreed that children's peers play a fundamental role in the type and quantity of food children eat (Table 3; QI, QII).

**Table 3.** Supporting quotations: theme ‘Social influences on children's eating behaviours’.

Subtheme	Quotation no.	Supporting quotation
*Peer influence*	I	Or say one child does not like eating carrots and his friend has put carrots on his plate, they might put carrots on his plate but might not choose to eat them or might just try one but not actually like them. So I think it is a lot to do with peer pressure. And as well, at the table, something they might never eat, but if everyone at that table is eating they might then eat the plate, which has kind of surprised us. [Nursery staff 1, F, FGD 3]
	II	Yes, and she's [daughter] asking for that when she comes home, whatever these other kids have had in their lunchbox, so it's all about that person had that in their lunch. Why can't I have it? [Parent 3, F, FGD 4]
*Grandparent influence*	I	I think more and more grandparents are playing a big role in childcare. And… But my mum, I’ve definitely noticed she has more sweets, more biscuits, more… because I’m really… I’m definitely careful about that […] And whenever she [participant's mother] goes and looks after him [participant's nephew], she doesn’t always follow what his mum tells her. [Nursery staff 7, F, FGD 1]
** **	II	Grandads in particular, yes, seem to have forgotten everything about how they brought their own children up, and they’re the treat provider. […] They probably have the same size of first course, but they definitely have larger desserts, and we don’t always have dessert, […] but we do always have dessert when grandparents are around. Even at our house, they bring the dessert. [Parent 4, F, FGD 4]
*Nursery staff influence*	I	If the staff sit with the children […] and just have a little something and a little chat […] they’re eating better. And rather than, you know, just standing and cleaning plates and things, just taking that time. They don’t feel rushed, they’re having a bit of a chat. […] When I had a chat with her, she refused her meal, and I sat next to her and I had just a little bit. Oh, that's nice. You have a bit of your… and the next thing, she cleared her plate. [Nursery staff 8, F, FGD 1]
	II	[…] in our setting, we sit down with the children sometimes, while they are eating lunch, with a plate ourselves. So we are not just sitting there while the children are eating. More like what you would get at home, the experience. So while the child is speaking to you, you might be sitting with them, see they are not touching any of their vegetables, oh, have you tried your peas? And they might go oh, no I have not, but you’re eating yours. […] So it is just a bit of encouragement without having to say anything directly to encourage them. And again if they then don’t take it from that, then the encouragement, oh, these peas are good. And they will sort of think, mmm, and then they will try that. I think that affects how much they are eating and well they are willing to give it a try anyway. That does have a good impact. [Nursery staff 1, F, FGD 3]

##### Grandparent influence

Grandparents were identified as a barrier in the provision of age-appropriate PSs and healthful foods. Parents and nursery staff often suggest that grandparents counteract the pre-existing portioning and feeding practices instilled in children (Table 3; QI, QII).

##### Nursery staff influence

Nursery staff described how they influence the amount of food children consume by using feeding practices, such as encouragement, to persuade some children to finish their portion and try new foods (Table 3; QI, QII).

### Theme 4: Child-specific, social and external factors influencing parent and nursery staff PS decisions

#### Child-specific factors

##### Appetitive characteristics of the child

Widespread belief existed amongst parents and nursery staff that children's appetitive characteristics are unique and an appropriate PS for one child could be excessive or insufficient for another (Table 4; QI, QII). Parents and nursery staff mentioned children's appetitive characteristics can change, for example during a growth spurt, and often parents noted that children eat more (Table 4; QIII) and eat less based on their emotional state (Table 4; QIV). Under such conditions, some parents were aware of the need to be more responsive than prescriptive (Table 4; QIII).

**Table 4.** Supporting quotations: theme ‘Child-specific factors influencing parent and nursery staff portion size decisions’.

Subtheme	Quotation no.	Supporting quotation
*Appetitive characteristics of the child*	I	I think every child's different. You know, as you were saying about physical exercise or, you know, the routines of the day, the sleep patterns […] If you say the government says, you should have this amount or that amount, you’d say, well that doesn’t work for our kid here. […] I think you have to just deal with what's in front of you at that particular time. [Parent 1, M, FGD 4]
	II	[…] you learn your kids, like you know your kids, you know how much they need, how much they don’t need, and I think that's kind of maybe the guide. [Parent 3, F, FGD 4]
	III	Yes, I think they [children] go through growth spurts, so I think they go through extreme bouts of angry hungriness, and I think you just have to roll with it for that week, and then it goes back to a dull roar. […] I think, really, you can tell that they’re kind of gearing up for a massive growth. So I guess sometimes I’m a bit more relaxed, and I’ll let it. [Parent 6, M, FGD 4]
	IV	If they’re [children] stressed about something, or something's happened that's upset them, or they’re tired, generally, they won’t eat very much. [Nursery staff 6, F, FGD 1]
*Age of the child*	I	Things like fishfingers and that, the older children get more than maybe the younger ones. [Nursery staff 4, F, FGD 1]
** **	II	Like, our older ones [children] know when to stop. They’ll be, like, I’m full, I don’t want this. [Nursery staff 6, F, FGD 1]
*Activity level*	I	Yes, but I think that on my days with the children, I will have in my head how much they’ve eaten and how much exercise they’ve done. And maybe that will subconsciously make me give them bigger portions in the evening. Because I’d know, oh, we’ve had a really busy day. We’ve been on our bikes, we’ve walked miles, you know. [Parent 3, F, FGD 2]
** **	II	Well in our room there might be certain children that are just generally more hungry, maybe because they are a little bit more active children. So they tend to be a lot more hungry and maybe ask for more snacks. Whereas you know there are certain children that don’t run around so much or on bikes, they might not ask for as much snacks. So you are kind of aware when how much portion they actually ask for. [Nursery staff 1, F, FGD 3]

##### Age of the child

Consensus existed amongst nursery staff that child age is an influential factor in determining PS, with older children tending to receive larger PSs than younger children (Table 4; QI). Furthermore, some nursery staff perceived older children to be better at self-regulating their food intake than younger children (Table 4; QII).

##### Activity level

Parents and nursery staff mentioned the activity level of children affects the PS served, with some being inclined to serve larger PSs if children have had an active day (Table 4; QI, QII).

#### Social factors

##### Partner/parental influence

Several parents revealed decisions are often made jointly with their partner about appropriate PSs. One parent identified their partner as an important facilitator in the provision of age-appropriate PSs given their past occupation (Table 5; QI). Parental portioning and feeding practices often differed from their partners, with some suggesting these differences stem from their upbringings (Table 5; QII-QIV). Some parents expressed not wanting to subject their child to the authoritarian and restrictive portioning and feeding practices they experienced themselves (Table 5; QV). Nursery staff also mentioned how feedback from parents can influence their portioning practices (Table 5; QVI).

**Table 5.** Supporting quotations: theme ‘Social factors influencing parent and nursery staff portion size decisions’.

Subtheme	Quotation no.		Supporting quotation
*Partner/parental influence*	I	I check with my husband. He used to be a chef, so he did training in portion sizes for kids. Yes, so sometimes if I think, not sure, it's maybe a bit much, I say to him, what do you think? Yes, he tends to say, it is a little bit too much. [Parent 4, F, FGD 4]	
	II	I’ve noticed a really big difference between my partner's attitude to food and snacks than mine, and it's definitely come from our upbringing. So I grew up on a farm, where food is available 24 hours […] But when I see my kids at my husband's parents’ house, it's very different […] less grazing, more structured. […] he's [partner] concerned that our girls probably eat too much. But I don’t think they eat too much. [Parent 7, F, FGD 2]	
	III	Yes, so my husband and I differ in parenting skills […] because I think that they [children] can decide, and if she [daughter] doesn't want to eat, that's fine, because she doesn't get treats. So that's kind of the compromise, it's the decision they have to make, whereas my husband wants them to finish their dinner, regardless if they’re really not keen on it. So sometimes it's an hour long, sitting at the table. [Parent 3, F, FGD 4]	
	IV	But I think part of it is [name of wife's] parents, my wife's parents, were very old-fashioned in that regard. Like that is exactly what you get, and you will not deviate. If you eat it, great, and if you don’t eat it, you will be hungry. And I guess we’ve kind of taken that pretty stern view. [Parent 6, M, FGD 4]	
	V	And it's actually my mum who, I think, when we were kids, they were quite strict about what we ate and how much we ate. And so when me and my brother were teenagers and went off to university, we both went bonkers about food […] just because we hadn’t […] had it as kids growing up. […] So I’ve gone down the road of […] they can have chocolate or they can have crisps, but it's in moderation. So it's trying to get them to know that you can have chips, but you need to able to, like, stop at some point, which is something I need to learn. [Parent 2, F, FGD 2]	
	VI	Like we would do like a hand over with the child's parent in the morning, the parent might say they [the child] […] didn’t have a breakfast then we are aware of that, that that child might be more hungry come snack time. And say they might only be allowed usually a slice of toast, we would then know if that child did want more they can come and ask or we’d say would you like any more if the child is hungry. [Nursery staff 1, F, FGD 3]	
*Sibling influence*	I	Ours [children] are fiercely competitive. So if [name], the eldest, he gets two sausages, then both [child 2 name] and [child 3 name] want two sausages each, and they’ll be quite angry when I give them one sausage, right? But the fact that, oh, it's not fair that's a big competition issue in our house. [Parent 6, M, FGD 4] We have that too, actually. There's just over a year between them [their children], and we’ve always treated them the same, but the older one's going through a phase where she needs more food… and the younger one is not happy, because she is not also getting more food, yes. […] She shouldn't be having more food. She doesn't need more food. She just wants it because her sister's having more. [Parent 4, F, FGD 4]	
** **	II	[…] there's six years between them, so I don’t think I really give my elder the same size or portion as my younger son, and he wouldn’t say to the younger one, ah-ha, I’ve got more than you. And the younger one wouldn’t say, well, he's got more than me. […] but I think that's maybe because there is quite a gap there. [Parent 2, F, FGD 4]	

##### Sibling influence

Some parents expressed their difficulty in helping their younger children comprehend why they require smaller PSs compared with their older siblings, with some admitting this caused great conflict within the household (Table 5; Q1). Contrastingly for other parents, the presence of older siblings had no influence in portioning and feeding practices (Table 5; QII).

#### External factors

##### Past experience

When judging appropriate PSs, some parents and nursery staff expressed they are usually a result of guess-work or intuition often gained through previous experience (Table 6; QI-IV).

**Table 6.** Supporting quotations: theme ‘External factors influencing parent and nursery staff portion size decisions’.

Subtheme	Quotation no.	Supporting quotation
*Past experience*	I	[…] everything [portion size decisions] just comes over time. [Parent 3, F, FGD 2]
	II	Well, we had a bit of trial and error as well, in the sense that, you know, we could pour out the bowl, and as you were saying, you know, an hour later, oh, I’m hungry, I’m hungry. [Parent 1, M, FGD 4]
	III	You know, you just eyeball it [portion sizes]. [Parent 6, M, FGD 4]
	IV	We just judge it [portion sizes] ourselves. [Nursery staff 4, F, FGD 3]
*Tableware*	I	I use smaller plates, like plastic plates, for, like, his size […] And then just kind of fill up that. [Parent 2, F, FGD 2]
	II	The size of the plate probably has something to with it, because you probably do it without realising. If you have a bigger plate, you’re probably going to put more food on it. [Nursery staff 5, F, FGD 1]
	III	The cutlery is child sized and so are the plates and the bowls. [Nursery staff 5, F, FGD 3]
	IV	Well, if there is cereal, they’ll have a ladle. So they [children] would put one scoop into the bowl. [Nursery staff 1, F, FGD 3]
	V	You get some of the parents that use the wee square tubs. [Nursery staff 5, F, FGD 3]
	VI	But when you look at them [portioning tubs] sometimes, you’re just like, that's tiny. But then we’re looking at it from an adult's… You know, we’re both looking at it with an adult's eyes, going oh my god, that would never fill me up. [Nursery staff 6, F, FGD 1]

##### Tableware

Several parents and nursery staff described child-sized tableware, such as plates, bowls, as facilitators in determining appropriate PSs (Table 6; QI-QV). One nursery staff noted, from an adult's perspective, food portioning tubs appear too small (Table 6; QVI).

### Suggested useful tools/strategies in PS decisions

Some parents and nursery staff provided suggestions for tools/strategies they would find useful in determining PS. These included: different sized ladles, standardised bowl sizes for different age categories, image analysis software, and food subscription services tailored to the PS requirements of young children (Table 7; QI-III).

**Table 7.** Supporting quotations: ‘Suggested useful tools/strategies in portion size decisions’.

Quotation no.	Supporting quotation
I	[…] just having standardised bowl sizes for different age ranges might be a worthwhile thing. [Parent 5, M, FGD4]
II	[…] you can imagine, with image analysis […] You know take a photo of it, and it says, yes, that's too much potato, and people will see, you know. [Parent 6, M, FGD4]
III	But one could imagine HelloFresh [food subscription service] or similar providers doing something where they also had portion control for children, because at the moment it's entirely for adults. […] And they tell you calorie information and they it put only for adults. So you could imagine a kind of pre-packaged solution for busy parents that was a bit more friendly. [Parent 6, M, FGD4].

## Discussion

This study explored parent and nursery staff perceptions and influences on child PS provision. Most parents and nursery staff held similar opinions on PS perceptions and influences, but notable differences were found between parents and nursery staff on the awareness of PS guidelines; nursery staff were less aware of existing PS guidelines than parents.

Our findings suggested that awareness of PS guidelines was mixed for both parents and nursery staff. Although some nursery staff were vaguely aware of the existence of PS guidelines, they were unaware of what the guidelines consisted of, a concern given their increased role in the selection and provision of child food PSs (Gubbels et al., 2010; Larson et al., 2011). While some parents had an awareness of guidelines, they often admitted disregarding these due to the effort involved in weighing out food (Croker et al., 2009; Curtis et al., 2017; Reale et al., 2019a; Tang et al., 2020). Instead, parents expressed that PS judgements are normally a result of guess-work (often ‘eyeballing’ it) or intuition gained through experience (Blake et al., 2015; Croker et al., 2009; Curtis et al., 2017; Eck et al., 2018; Jacquier et al., 2016; Johnson et al., 2015), suggesting the use of tacit rather than explicit knowledge when determining children's PS. The provision of further information/guidance on food portioning was welcomed by nursery staff, supporting other caregiver research (Eck et al., 2018; Martin-Biggers et al., 2015; Sherry et al., 2004). Thus, it may be beneficial to educate nursery staff on PS guidelines regularly via practical and interactive training, including at enrolment. For parents, providing easily accessible and visual PS guidelines and prompts may be useful to remind parents to provide age-appropriate portions, and help ensure that their own ‘adult size’ portion and/or elevated portion norm is not mirrored in the portion they serve for their child (Johnson et al., 2014; Nicklas et al., 2013; Reale et al., 2019b).

We found variation in the extent of autonomy granted to young children by parents and nursery staff. Nursery staff disclosed that granting young children autonomy over food choice/PS is a relatively new practice endorsed by the early years sector (Public Health Scotland, 2018), which has had positive implications, such as encouraging young children to practice self-service and responsive eating. Providing encouragement and nutrition education for young children could be used in conjunction with granting autonomy to help support them in developing healthy eating choices. When actively involved in food preparation and/or selection, some parents mentioned that children were more inclined to consume the meal without fuss. The combination of these findings supports evidence of the benefits of engaging young children in PS selection and food preparation (Anliker et al., 1992; Chu et al., 2014; Van der Horst et al., 2014).

The perceived healthfulness of food often determined the extent of control exerted by some parents and nursery staff, whereby foods deemed ‘healthy’ are restricted less compared with foods deemed ‘unhealthy’ (Blake et al., 2015; Carnell et al., 2011; Croker et al., 2009; Eck et al., 2018; Fisher et al., 2015; Jacquier et al., 2016; Petrunoff et al., 2014; Sherry et al., 2004). Restriction of foods, particularly HED foods, in childhood can result in adverse eating outcomes later in life (Jansen et al., 2007). Therefore, downsizing HED portions and offering large portions of low-energy-dense foods (e.g. fruits and vegetables) could be an effective strategy in promoting/encouraging appropriate portion control in young children (Carstairs et al., 2018; Reale et al., 2018). A common strategy for controlling PS by caregivers in our study was to provide children with smaller PSs and permit second helpings if children were still hungry (Croker et al., 2009; Infant & Toddler Forum, 2014). This portioning strategy permits input from both the caregiver and the child and may encourage young children to eat in response to internal cues of hunger and satiety rather than external cues.

Individuals often model the behaviours of others they consider themselves most alike and with whom they frequently interact (Bandura, 1977). Consistent with social learning theory and literature on peer influences on child eating behaviours (Higgs and Thomas, 2016; Houldcroft et al., 2014; Petrunoff et al., 2014; Salvy et al., 2012), peers were perceived as important role models in the development of a child's eating behaviour by parents and nursery staff. As more children are attending full-time non-parental childcare (Care Inspectorate, 2022), and consuming most of their daily meals in these environments with their peers (Gubbels et al., 2010; Larson et al., 2011), the nursery setting is therefore an important consideration for portion influence and consumption behaviour in children. Potential challenges could exist where unhealthy eating norms prevail among peers and thus is something for nursery staff to be cognisant of. Our findings also show that in this setting, nursery staff can influence the amount of food children consume, primarily through communication-related feeding practices, such as verbal encouragement (Dev et al., 2016; Love et al., 2020; Ramsay et al., 2010). While responsive/healthful communication-related feeding practices can have positive implications, such as assisting with acceptance of new foods (Blissett and Fogel, 2013), controlling/restrictive feeding practices can have negative implications, such as encouraging young children to neglect internal cues of hunger and satiety (Batsell et al., 2002; Birch et al., 2003; Carper et al., 2000; Galloway et al., 2006; Johnson, 2000). Educating food providers about the difference between responsive/healthful and controlling/restrictive communication-related feeding practices may prove beneficial in promoting age-appropriate food portion consumption. A novel finding of the current study was the positive influence of parental feedback and communication on the portioning practices of nursery staff. Therefore, engaging parents in discussions about their child's consumption patterns and behaviours might be of value for nursery staff in their role as food provider.

According to parents and nursery staff PS decisions were often based on the individual child's appetitive characteristics (Carnell et al., 2011; Croker et al., 2009; Jacquier et al., 2016; Johnson et al., 2015; Petrunoff et al., 2014; Reale et al., 2019a), such as satiety responsiveness. There was acknowledgement that children's appetitive characteristics can change due to factors such as developmental stage or emotional state, and under such conditions, there is a need to be more responsive than prescriptive, particularly during periods of growth. We found consensus amongst parents and nursery staff that child age and activity level are influential factors in determining PS. Both parents and nursery staff indicated that older children receive larger PSs than younger children, matching child PS guidelines (Infant & Toddler Forum, 2014), suggesting some parents and nursery staff may unknowingly follow age-related portion control. Additionally, older children were observed as better at self-regulating their food intake compared with younger children by nursery staff. This supports experimental research suggesting age-related differences in children's response to the PS effect (Fisher, 2007; Fisher and Kral, 2008; Mathias et al., 2012). Larger PSs were served to children following an active day by both parents and nursery staff. Although this suggests caregivers consider the energy balance of children, research has found that parents often overestimate children's activity levels (Kesten et al., 2015; Lau et al., 2013) and therefore energy expended, and this might lead to overconsumption. Improving parent and nursery staff knowledge of the concept of energy balance in young children via educational interventions may be beneficial. Personalised guidance on how parents and nursery staff should respond to children's unique appetitive characteristics could also be useful in the interpretation of child needs. While research has been conducted on interpreting appetitive cues in infants (Hetherington, 2017, 2020), similar research has yet to be conducted in pre-school aged children.

Parental portioning decisions often were made jointly with partners (Curtis et al., 2017), some were influenced by parental upbringing and a desire to avoid replicating the authoritarian/restrictive feeding practices they experienced as a child (Eck et al., 2018; Petrunoff et al., 2014). Grandparents were identified as a barrier to the provision of age-appropriate PSs and healthful foods; the same people who as parents were restrictive over unhealthful foods now place less restriction on these food items for their grandchildren. This finding is unsurprising as research suggests grandparents often use unhealthful HED foods to convey love and encourage children to consume more food than physiologically required (Curtis et al., 2017; Faith et al., 2012; Jingxiong et al., 2007; Lorentzen et al., 2012). Future research could focus on the acceptability of PS guidance to caregivers within the wider family household.

Child-sized tableware was often identified as a facilitator in the provision of age-appropriate PSs, supporting evidence indicating that the size and shape of tableware contributes to the amount of food consumed (DiSantis et al., 2013; Mishra et al., 2012). Both parents and nursery staff identified child-sized tableware as a useful PS tool (Curtis et al., 2017; Eck et al., 2018; Jacquier et al., 2016; Reale et al., 2019a; Tang et al., 2020), and providing parents with an easy, less time-consuming alternative to food weighing. Parents also suggested that image analysis software and food subscription services tailored to the PS requirements of young children may be beneficial and could assist with the provision of age-appropriate PSs. Future research could identify additional innovative tools and strategies that parents and/or nursery staff would find useful in daily PS decisions.

The primary strength of this study is the novel contribution made to the limited existing qualitative evidence-base exploring both parent and nursery staff perceptions and influences on child PS. However, the findings from this purposive sample should be interpreted with caution and may not be generalisable, as they primarily reflect the perspectives of female parents and nursery staff living in Scotland. Therefore, the identified perceptions and influences are less representative of fathers, male nursery staff and other caregivers and those living outside of Scotland. This study lacks some additional demographic insight. As PS consumption behaviour is influenced by socio-demographic factors (Piernas and Popkin, 2011; Yamoah, 2019), future research should explore whether socio-demographic factors, such as age, gender and income, have an impact on parent and nursery staff PS selection and provision for young children aged 3–5 years.

To our knowledge, this is the first qualitative study to investigate both parent and nursery staff perceptions and influences on child PS. Our findings suggest the extent of caregiver control and social, child-specific and external factors are more influential than PS guidelines in parent and nursery staff PS selection and provision for the young children in their care. Although most parents and nursery staff held similar opinions on PS perceptions and influences, a key difference found was in their awareness of PS guidelines. Another key message from this study was that engagement between children and caregivers is paramount in encouraging age-appropriate PSs and healthy PS norms. The findings can inform future PS educational and promotional campaigns and policies targeting caregivers of young children. Acknowledgement by Government, local authorities, educational institutions and families of the importance of appropriate portion control in the dietary habits of young children is key. Providing support and opportunity to implement healthy choices and age-appropriate portion provision is required. For example, regular PS training could prove beneficial for nursery staff caregivers. This could be aligned with the same consideration currently applied to food safety and hygiene. Training could be offered to all staff members annually and supported by Government and local authorities. Active, acceptable and effective communication of user-friendly PS information and guidance to both parents and nursery staff must be considered to aid effective portioning practices appropriate for age and stage. Innovation in food packaging and labelling could be utilised to support interventions for best practice in portion provision and could be a focus for future co-design research involving key stakeholders. By providing caregivers with the knowledge and tools to implement age-appropriate portioning practices, this learning could be utilised to educate and support children about healthy eating habits.

## Supplemental Material

sj-docx-1-nah-10.1177_02601060241245255 - Supplemental material for ‘You just eyeball it’: Parent and nursery staff perceptions and influences on child portion size: A reflexive thematic analysis Supplemental material, sj-docx-1-nah-10.1177_02601060241245255 for ‘You just eyeball it’: Parent and nursery staff perceptions and influences on child portion size: A reflexive thematic analysis by Sophia Quirke-McFarlane, Sharon A Carstairs and Joanne E Cecil in Nutrition and Health

sj-docx-2-nah-10.1177_02601060241245255 - Supplemental material for ‘You just eyeball it’: Parent and nursery staff perceptions and influences on child portion size: A reflexive thematic analysis Supplemental material, sj-docx-2-nah-10.1177_02601060241245255 for ‘You just eyeball it’: Parent and nursery staff perceptions and influences on child portion size: A reflexive thematic analysis by Sophia Quirke-McFarlane, Sharon A Carstairs and Joanne E Cecil in Nutrition and Health
