# Supplementary material for: ‘You just eyeball it’: Parent and nursery staff perceptions and influences on child portion size: A reflexive thematic analysis
Source: Nutr Health. 2024 Apr 16;31(2):701–14. doi: 10.1177/02601060241245255 (PMC12174632; doi:10.1177/02601060241245255)
Supplement: sj-docx-1-nah-10.1177_02601060241245255 - Supplemental material for ‘You just eyeball it’: Parent and nursery staff perceptions and influences on child portion size: A reflexive thematic analysis [file sj-docx-1-nah-10.1177_02601060241245255.docx]

Supplementary Table 1. *Discussion topic guide for the focus group discussions*

| Procedure | Summary of Procedure |
| --- | --- |
| Introduction | Provide information sheet and consent form for participants to read and sign  Introduce the beginning of digital recording  Re-iterate that the discussion will remain confidential  Introductions/ice-breaker:  *Parents*: Can I please ask you all to tell me a little bit about you and your family: how many and what ages of children?  *Nursery staff*: Could I ask you all to tell me a little bit about you and your experience in childcare? |
| Weighing task | Can I please ask you to serve a portion of cereal that you would provide to your child/the children aged 3-5 years under your care? |
| Exploration of perceptions and influences on child portion size | How do you decide how much of a food you give to your child/the children?  *Probes*: Does your child/the individual child play a role in this decision?; Are second servings offered/requested? What response is given to request for second helpings?; Do other people have a role in the portion sizes your child is given?*  *Prompts*: Pre-served cereal portions; Child response to portions; Plates, tools, etc.; Peers; Siblings*; Portion sizes for meals (differ across meals?)*; Snack portions*; Role of pre-packaged foods (sizes and price)*  Can you think of any situation where the amount you give your child/the children changes?  *Prompts*: Who they are with; Eating out/away from home*; Their mood/health*; Type of food**  Do you feel you know how much your child should be getting?*  Are you aware of any recommendations for food portion sizes for children?  *Probes*: Where do you get your guidance on portion size selection? Do you think this guidance is helpful?*; Do you have specific guidance on portion size selection? Do you think this guidance is helpful?**; Have you attended/received any training on portion size provision?**  Do you have any concerns about the amount of food that your child/the children under your care eat/receive?  Are there any objects/strategies that you use or think could be helpful in selecting appropriate portion sizes? |
| Finishing up | Are there any other influences that effect the portion size your child/the children under your care receive that we have not discussed?  “Thank you for your participation in this discussion” |

*Note.* * Questions, probes and prompts asked to parents only; ** Probes and prompts asked to nursery staff only.
