# Supplementary material for: ‘You just eyeball it’: Parent and nursery staff perceptions and influences on child portion size: A reflexive thematic analysis
Source: Nutr Health. 2024 Apr 16;31(2):701–14. doi: 10.1177/02601060241245255 (PMC12174632; doi:10.1177/02601060241245255)
Supplement: sj-docx-2-nah-10.1177_02601060241245255 - Supplemental material for ‘You just eyeball it’: Parent and nursery staff perceptions and influences on child portion size: A reflexive thematic analysis [file sj-docx-2-nah-10.1177_02601060241245255.docx]

Supplementary Figure 1. Identified themes and subthemes derived from the detailed thematic analysis of the four focus group discussions

*
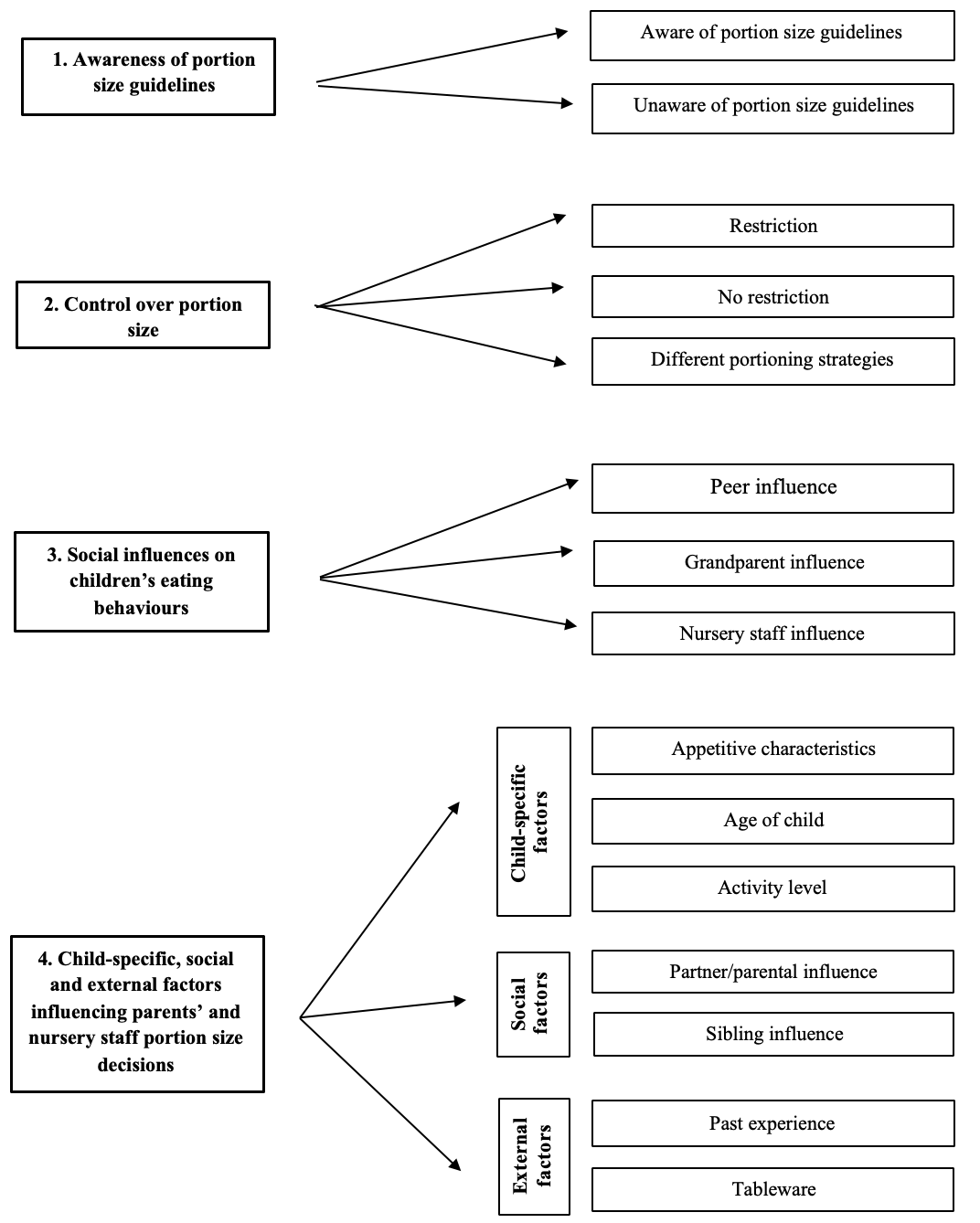
*
